# Corporate Business Strategy and Tax Avoidance Culture: Moderating Role of Gender Diversity in an Emerging Economy

**DOI:** 10.3389/fpsyg.2022.827553

**Published:** 2022-05-27

**Authors:** Xiaochen Zhang, Muhammad Husnain, Hailan Yang, Saif Ullah, Jaffar Abbas, Ruilian Zhang

**Affiliations:** ^1^Business School, Hohai University, Nanjing, China; ^2^Department of Business Administration, University of Sahiwal, Sahiwal, Pakistan; ^3^Business School, Shandong Jianzhu University, Jinan, China; ^4^Lahore Business School, University of Lahore, Lahore, Pakistan; ^5^School of Media and Communication, Shanghai Jiao Tong University, Shanghai, China; ^6^Antai College of Economics and Management, Shanghai Jiao Tong University, Shanghai, China; ^7^Research Center of Social Risk Assessment, School of Public Administration, Hohai University, Nanjing, China; ^8^Centre for Social Responsibility in Mining, Sustainable Minerals Institute (SMI), University of Queensland, Brisbane, QLD, Australia

**Keywords:** business strategy, tax management, gender diversity, tax avoidance, GMM model

## Abstract

Tax payments stimulate business enterprises to choose tax management through tax avoidance activities, which is the legal practice to reduce the amount of tax payable. In developing economies, taxation is considered more critical for budget and revenues of a country. This paper investigates whether various business strategies influence corporate tax avoidance decisions of firms by adopting business strategies. Besides, it explores how gender diversity can ease this relationship. This study has chosen a sample of organizations from non-financial sector in Pakistan. The time frame is 5 years, including once a year. The present model employed a generalized moment method (GMM) and tested the proposed hypothesis to draw the results. The study has taken the size, leverage, and business profitability as control variables of firms. The study outcomes by using the GMM method demonstrate that the presence of female directors reduces tax avoidance behavior in prospector companies. This study provides insight into future research for stakeholders, government officials, tax authorities, and policymakers. The findings offer valuable recommendations and practical insights and implications. The findings provide future directions for research to test different frameworks to attain beneficial results to promote the responsibility of tax payment culture.

## Introduction

The maximization of a firm’s value is the main objective of business decisions (Abbas et al., 2021; Mubeen et al., 2021b; Wang et al., 2021; Li et al., 2022; Liu et al., 2022). The top management takes favorable decisions to enhance firm performance (Abbas et al., 2019c; Hussain et al., 2019, 2021; Mamirkulova et al., 2020; Mubeen et al., 2021a; Wang et al., 2021). Organizations emphasize social good and create a sense of social reasonability to contribute to society’s welfare (Abbas et al., 2019b,^2020^; Mubeen et al., 2020; Abbasi et al., 2021b; Aman et al., 2021). Organizations incorporate innovation, knowledge sharing, and corporate social responsibility (CSR) practices to maximize profitability (Hussain T. et al., 2017; Abbas et al., 2019a,b; Azadi et al., 2021; Azizi et al., 2021). If a transaction for any period increases tax cost, it shows cash outflows; otherwise, it will result in cash inflow and represent tax savings. The tax savings must be higher than tax costs to increase the value of a firm (Gokten and Kucukkocaoglu, 2018). The ability of firms to pay low taxes for a more extended period is referred to as tax management (Minnick and Noga, 2010). Companies used to be inclined to commit to tax planning when they supposed it to be an activity that could increase their value. According to Minnick and Noga (2010), although the management of taxes can cause an improvement in the bottom line of a company, it is also essential to know that there is present an association of costs with the selection of investing the resources in tax planning.

Tax management is essential for the growth and profitability of a company (Shackelford and Shevlin, 2001). The term tax avoidance is a situation where companies execute specific tax-related policies. There is a probability that tax measurement will not be interrogated on a legal basis. Fadhila and Handayani (2019) describe tax avoidance as an evasion effort legally conducted for taxpayers because it does not entail taxation provisions. According to Gokten and Kucukkocaoglu (2018), tax avoidance is a legitimate means of minimizing taxes. Tax avoidance is a way of avoiding taxes or reducing tax amounts, and it refers to using laws regarding tax in a manner that is not proposed by the government (Hoseini and Safari Gerayli, 2018).

Previous studies define prospectors as innovative firms seeking new products and new market opportunities. They have less focus on minimizing costs and are more concerned about experimentation and innovation. Therefore, prospectors are budget-oriented toward marketing and Research and Development (R and R&D) activities and act toward developing various technologies for the diverse nature of their product mix (Bentley et al., 2013; Hsu et al., 2018). In prior literature, the reason behind the behaviors of firms toward engaging in different degrees of tax avoidance is also not apparent. Business Strategy may differ in a single industry. Thus, if there present a relation between tax avoidance and business strategy, it can clarify the inter-industry variation in the tax avoidance activities of a firm (Koester et al., 2017). Dunbar and Phillips (2001) had categorized the companies as defenders or prospectors. They studied the relationships between these two business strategies and the outsourcing of the corporate tax function activities. Previous empirical and theoretical research on management suggests business strategy as a multidimensional variable, but it can be differentiated based on acting as a single variable (Miles et al., 1978; Hambrick, 1983).

Corporate governance plays an essential role in monitoring different actors and harnessing planning procedures. In this perspective, numerous studies (Desai and Dharmapala, 2006; Hanlon and Slemrod, 2009; Chen et al., 2010; Lanis and Richardson, 2011) have shown that corporate governance adversely affects tax aggressiveness. To investigate it further, Pratama (2017) reveals that a wide range of firm characteristics, such as age, size, and earnings, create a considerable impact on tax avoidance strategies. Corporate governance proxies like audit firms, audit quality boards, and commissioners’ sizes also affect tax avoidance.

Female directors on board are also among the characteristics that must be considered as firms’ board attributes. The existence of women on the board is thought to be associated with tax avoidance (Lanis and Richardson, 2015; Hoseini and Safari Gerayli, 2018). In this perspective, Richardson et al. (2016) described that a female director’s presence could increase a firm’s monitoring function. In this manner, the companies need to pay attention to the proper board of directors’ composition to minimize tax avoidance. Contrary to this, Hoseini et al. (2019) concentrated on the fact that female directors can benefit companies in making decisions and policies regarding tax avoidance.

Although an abundant amount of present literature has focused on connecting a company’s multiple features with tax management. Past literature considering the agency roles (Chen et al., 2010; Minnick and Noga, 2010; Armstrong et al., 2015), literature relates tax avoidance with compensation (Desai and Dharmapala, 2006; Minnick and Noga, 2010; Rego and Wilson, 2012), ownership (Chen et al., 2010; Steijvers and Niskanen, 2014; Moore et al., 2017), and the institutional ownership (Chen et al., 2010) along with other corporate governance-related traits.

This study focuses on the female directors on the board as moderators and how prospectors’ business strategies can influence a company’s decision regarding tax avoidance. Gender diversity as a characteristic of corporate governance in executive ranks can also affect the manager’s behaviors (Zemzem and Ftouhi, 2013; Arun et al., 2015; Boussaidi and Hamed, 2015). However, several other researchers have also found that gender diversity in boards has no link with tax planning and tax aggressiveness (Khaoula and Ali, 2012; Francis and Ganeshamoorthy, 2017). This gap in the research literature has inspired this study to scrutinize the board size and the existence of female directors on tax avoidance by firms. Furthermore, the link between tax avoidance and business strategy while taking corporate governance mechanisms as moderators is not found. Hence, there is a need to extend the tax avoidance literature concerning its influencing capacity to effected by the business strategy adopted by the firm’s governance in developing economies like Pakistan. We contribute to the literature in the following ways. First, it would contribute to the business strategy literature by investigating the role of business strategy tax avoidance.

Second, this study extends the work by Hsu et al. (2018) by analyzing the business strategy’s influence on tax avoidance while using the business strategy typology given by Miles et al. (1978) and using corporate governance mechanisms acting as moderators. This study also has policy implications. Taxation is critically essential in strategic business decisions, and as a result, firms are considering a dynamic and proactive approach to tax administration. Corporate tax evasion operations are seen as activities that add value to the stock price of a company and boost its market capitalization. According to the conclusion of the study, each organization should have a set optimal level of tax avoidance or tax planning that can balance the benefits and costs of tax avoidance and plan in a way that maximizes shareholder value. It also helps authorities see that tax aggressiveness can impact shareholder returns that are linked to investment decisions, as well as on management, who may benefit from tax savings, which is favorable to them. As a result, tax evasion has become a worry for society, the government, and other authorities and decision-makers. As a result, public-private partnerships are more aggressive in their tax planning.

## Theoretical Background and Hypothesis Development

This section describes the theoretical background and hypothesis development of the study.

### Agency Theory

Management’s action is considered exclusively for reducing the taxes by setting tax-aggressive activities that are becoming common in all firms worldwide (Boussaidi and Hamed, 2015). Lanis and Richardson (2011) concluded that taxes act as the motivational factor for managers making decisions. There is a possibility that the companies’ aggressiveness in tax avoidance may necessarily not be consistent with the desires of shareholders of a firm, which represent a discrepancy among the wishes of the shareholders with the behavior of management which is commonly known as agency theory. According to Hoseini et al. (2019), agency theory is a theory that clarifies the relationship between the owner of a company (shareholder) and the management of the company, and in between them, management acts as an agent that is appointed by the shareholder (principals). The management is given the jobs, tasks, and authority for managing the firm, and it does it under the supervision of the shareholders (Herrera-Cano and Gonzalez-Perez, 2019). In practice, the principals desire to pay taxes by management in their actual amounts, while the administration wants to minimize the tax expenses for obtaining higher profits. Although, there are times when the interests of the management and shareholders are the same. In this way, the things that can boost tax avoidance can also be generated (Hoseini et al., 2019). Tax aggressiveness is determined by the nature and the agency conflict extent.

Many researchers call for additional studies for examining tax aggressiveness in the context of agency conflicts (Desai and Dharmapala, 2006; Lanis and Richardson, 2011; Scholes et al., 2014). Desai and Dharmapala (2006) describe that when the volume of directors is more significant, the incentivized interests of directors will be higher, and the opportunity for tax avoidance will also be higher. Additionally, according to Ye et al. (2019), gender diversity in terms of agency problems can cause improvements in managerial monitoring, and it can result in better decision-making in terms of the reduction in agency problems. In this regard, many researchers have also stated that an individual or group of people’s domination in the decision-making process can also be affected by gender diversity. In this way, gender diversity can decrease the conflict of interest between the managers and the shareholders, which can reduce the problem regarding tax aggressiveness (Boussaidi and Hamed, 2015).

### Hypothesis Development

While any firm governance structure includes a comprehensive and complex set of relationships, institutional structures, and agreements, therefore, in terms of determining its relation to tax avoidance, we will concentrate on those mechanisms that have a close link with tax and strategic framework decisions.

#### Business Strategy and Tax Avoidance

Taxes are said to be deductions from cash flows available to the firms. In this way, the owners try their best to maximize wealth by using several tax avoidance practices so that the dividends can be suitable for distribution. Wilde and Wilson (2018) and Martinez and Ferreira (2019) define tax aggressiveness as it can affect shareholders’ returns; in this way, the managers’ available compensation can get their benefits from tax savings. According to Higgins et al. (2015), the previous studies on tax management (Dyreng et al., 2010; Armstrong et al., 2015) primarily concentrate on firm-level characteristics. For instance, size, capital intensity, firm foreign operations magnitude, and R and R&D. Miles et al. (1978) showed evidence of the association between tax planning and lower ETR (effective tax rates).

Tax avoidance behaviors’ have been affected by business strategies, and it depends on how each strategic type’s characteristics cause an affect the costs and benefits of tax planning (Higgins et al., 2015). Furthermore, Dunbar and Phillips (2001) state that the prospectors have less concentration on minimizing income tax costs. Thus, in this way, they will outsource more of the activities related to tax planning and tax compliance. Prospector-type firms concentrate more on innovation and development, and less concentration is on minimizing cost. In reality, defenders are often more inclined to try to avoid the complexities and costs that come with capturing an aggressive tax position in the first place. Otherwise, prospector companies bear the risk and are trying to cope with the unpredictability in their tax planning (Sadjiarto et al., 2020).

Firms that concentrate on improving and developing new ideas are prospectors, and they may pay more taxes. At least one director is present from the audit committee and a finance expert (Hsu et al., 2018). However, Martinez and Ferreira (2019) have found that the business strategies of firms that influence tax management are not significant. Higgins et al. (2015) clarify that the tax avoidance potential is more effective in corporate prospectors than defenders. Various and diverse types of complexities characterize the developing nation’s environment, which is done because of the scarcity of resources and lack of predictability needed for the development in favorable ways.

The volatile environment in developing countries has produced an environment that ignores the business’s long-term planning. Therefore, in this way, when the developed world, which has promoted the theories about long-term planning and promoted and practiced them, faces a lot of intense obstacles when employed in emerging markets (Zamani et al., 2013; Parnell et al., 2015). The experiments and challenges for strategists of Pakistan are diverse because of the increase in the young population, natural richness, the uncertainties caused by the geostrategic importance and geopolitical importance of Pakistan, and the cultural dimensions in Pakistan. Thus, there is the possibility that the performance of firms and their strategic behavior and the highlighted assumptions might not grasp as accurately in the following environments (Anwar and Hasnu, 2016). The above research concludes leads to the following hypothesis:

H_1_: Business Strategy has a significant impact on tax avoidance in the emerging economy.

#### The Moderating Role of Gender Diversity

Another characteristics firms’ boards need to consider is female directors. Given the discoveries of Lanis and Richardson (2011) and other examinations by Adams and Ferreira (2009), female directors seem to have a comparable effect as far as being compelling screens undifferentiated from that accomplished by independent directors. #they inspect whether female board portrayal also affects tax aggressiveness and the extent of outside directors on the board. Richardson et al. (2016) clarified that the presence of female directors could improve the observing capacity of an organization. The presence of women on the organization board is additionally accepted to have a relationship with tax avoidance-related decisions (Lanis and Richardson, 2015; Hoseini and Safari Gerayli, 2018). Female directors have gotten much consideration regarding their active job in checking administrative execution. Female directors consistently put forth a valiant effort to adjust corporate conduct that is liable to society and shareholders (Prabowo et al., 2017). Kasirang et al. (2013) also expressed that women on-board can see hazards in different business associations and are warier than men.

Gender diversity in the ranks of executives impacts the behavior of managers (Zemzem and Ftouhi, 2013; Arun et al., 2015; Boussaidi and Hamed, 2015). Numerous other researchers also found that gender diversity in boards has no link with tax aggressiveness and tax planning (Khaoula and Ali, 2012; Francis and Ganeshamoorthy, 2017). The study by Hoseini et al. (2019) also finds that female directors can benefit the companies in making their decisions and policies to reduce tax avoidance practices. Baldry (1987) displays in terms of taxation that females are more likely to be compliant in the decisions regarding tax reporting than male board members. Ruegger and King (1992) studied that gender diversity in most cases is more significant in explaining the changes in ethical behaviors’ related to taxes.

Female directors onboard show trends toward risk averseness, and they have a high level of moral and ethical standards and exhibit their independent thinking. Thus, in this way, it is more sensible to imagine that the presence of female directors on the board can also significantly decrease the tax aggressiveness likelihood (Richardson et al., 2016). Fallan (1999) observes the attitude differences of gender differences based on the tax, and they find out that these differences in the attitude levels are being influenced by the amount of knowledge related to tax systems and the monitoring and mistake of board difficulties in a like method to the outside directors. Moreover, Richardson et al. (2016) surveyed 300 Australian companies and the data reports from 2006 to 2010. They examine the effect of the presence of women on the corporate boards in the reduction of tax avoidance. The outcomes of their research exposed that women on the corporate boards may have a significant impact on the reduction of tax avoidance. Srinidhi et al. (2011) examined 94 firms having female directors in board composition. The results showed that women in the board would significantly promote the transparency of the financial statements. The risk of corporate tax avoidance also decreases, which leads to falling in information asymmetry. By considering the above work regarding female directors on the board, this study hypothesizes about the moderator action of female directors on the board as:

H_2_: The association between business strategy and tax avoidance has been significantly moderated by the presence of female directors.

#### Control Variables

The research of Wahab and Holland (2012) shows that tax avoidance is also influenced by tax profitability. Until now, Richardson et al. (2013) demonstrate the contradictions in the relationships with the consequences of the influencing effects of tax avoidance. Because of the inconsistent research, it is hard to state whether ROA (profitability) is negatively or positively related to tax avoidance (Darsani and Sukartha, 2021). The return on assets (ROA) as a proxy for a company’s profitability has been demonstrated in numerous studies to associate with the equity turnover ratio (ETR) positively. When a firm attempts to avoid paying taxes, there is a positive correlation between organization (enterprise) and tax avoidance. Increased returns on assets (ROA) means that a company has a greater chance of situating itself in tax planning, resulting in a reduction in the amount of tax due. Dyreng et al. (2010) recommend that firm size play a role in tax management and track down that smaller companies have higher tax rates. Eddy and Angela (2020) that firms’ size factors significantly influence tax avoidance which implies that the larger the organization, the more prominent the assets possessed by the organization to deal with its taxation rate. The study by Minnick and Noga (2010) showed that firm size positively influences tax if the tax avoidance is GAAP ETR. However, there is no significant impact if the measure of utilization is cash ETR. Richardson et al. (2013) likewise showed no significant effect between size and tax avoidance.

The size of the company has an impact on tax aggressiveness as well as being a crucial factor of good governance. The total amount of resources claimed by the organization might be used to assess the size of the organization. It can be determined by translating the total assets into the normal logarithm of their value (Mubeen et al., 2021a; Mamirkulova et al., 2022). Leverage is a ratio used to measure the extent to which limit the firm assets is being financed by long-term debt. That describes how much a firm depends on external resources and funds compared to the internal resources and funds or the accumulated incomes to finance their assets. By getting in line with Richardson et al. (2016), the financial leverage can be measured by dividing the total debt by the company’s total assets, and it is hired as a control variable for research. Debt ratio (DER) is a proxy used for leverage. The high level of debt financing can also influence tax avoidance behaviors. However, few studies have displayed that leverage does not significantly impact tax avoidance (Minnick and Noga, 2010; Richardson et al., 2013). They found that tax avoidance, which GAAP-ETR can measure is positively affected by leverage. If the leverage ratio is more than the debt interest, the tax burden of the firm will be reduced. As a result, it seems possible to lessen the indicators of tax avoidance. According to the literature, firms with more leverage appear to pay less corporate taxes since they benefit from debt financing.

## Data and Methodology

The data of this research has been collected for the companies listed on the Pakistan stock exchange (PSX) for 5 years (2013–2017), with data from financial statements, official websites, and the Pakistan stock exchange webpage (Anjum et al., 2017; Hussain S.T. et al., 2017; NeJhaddadgar et al., 2020; Abbasi et al., 2021a; Local Burden of Disease HIV Collaborators, 2021; Paulson et al., 2021). The financial industry is omitted from this analysis since it has different criteria for debt financing and other business policies. Furthermore, we removed the firms having data is not accessible, ending in a total of 575 observations under consideration. GMM (generalized method of the moment) is used to estimate equations and test hypotheses.

### Measurement of Variables

This section explains the operational definition of the variables under consideration.

#### Tax Avoidance

Various measures capture the dependent variable (i.e., tax avoidance). Each action, however, has its limitations (Hanlon and Slemrod, 2009). The most frequently used and widely accepted measure in prior research is ETR (Dyreng et al., 2010; Higgins et al., 2015; Rudyanto and Pirzada, 2020). This research use GAAP-ETR for measuring tax avoidance. According to McClure et al. (2018), tax avoidance is calculated with the tax expense ratio with the pre-tax book income. GAAP-ETR is also known as accounting ETR in the US context and is also reported as per financial statements as ETR. GAAP-ETR basically reveals the collective proportion of accounting income payable as taxes. In this way, this measures the tax avoidance in relativeness with accounting earnings. It, therefore, measures tax avoidance relative to accounting earnings (Annuar et al., 2014).

This study uses the following formula to calculate GAAP ETR, which is utilized as a proxy for tax avoidance.


$$G\,A\,A\,P\,\_\,E\,T=\frac{T\,o\,t\,a\,l\,t\,a\,x\,E\,x\,p\,e\,n\,s\,e}{I\,n\,c\,o\,m\,e\,b\,e\,f\,o\,r\,e\,T\,a\,x}$$

#### Measurement of Business Strategy

**FIGURE 1:**
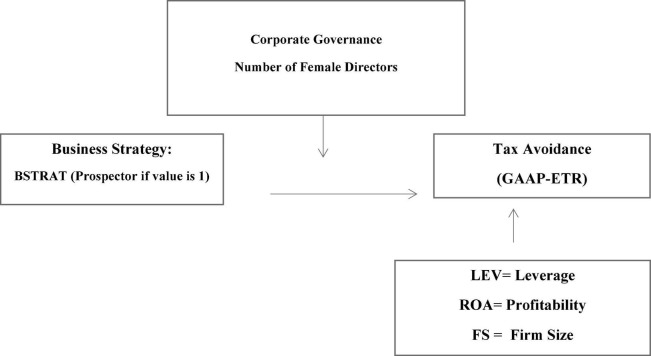
Theoretical framework of study.

By following the existing literature (Bentley et al., 2013; Higgins et al., 2015; Anwar and Hasnu, 2016; Hsu et al., 2018), we measure the business strategy as indicated in Figure 1, as given below.

##### Marketing Expense Ratio

The marketing expenses to sale ratio has been used (Hambrick, 1983; Bentley et al., 2013). It shows the tendency of organizations to innovate their operations. In respect to this ratio, it demonstrates that the corporation is concentrating its efforts on the latest commercialization developments. According to its results, it improves proficiency and splits the prospector and defender into groups (high for PROS and low for DEF).


$$M\,E\,S\,R=\frac{M\,a\,r\,k\,e\,t\,i\,n\,g\,E\,x\,p\,e\,n\,s\,e\,s}{S\,a\,l\,e\,s}$$

##### Cost of Goods Sold to Sales Ratio

The purpose of the COGSR is to know the concentration level of a company toward the internal efficiency that helps it lead to production efficiency (High for PROS and low for DEF) (Anwar and Hasnu, 2016).


$$C\,O\,G\,S\,R=\frac{C\,o\,s\,t\,o\,f\,G\,o\,o\,d\,s\,S\,o\,l\,d}{S\,a\,l\,e\,s}$$

##### Annual Sales Growth Rate

CASGR is a rate of changes that measures a firm’s orientation about its strategic growth (high scores denote PROS and low denote DEF) (Anwar and Hasnu, 2016).


$$C\,A\,S\,G\,R=\frac{E\,n\,d\,i\,n\,g\,v\,a\,l\,u\,e}{B\,e\,g\,g\,i\,n\,i\,n\,g\,v\,a\,l\,u\,e}-1$$

##### Operationalization of Business Strategy

**FIGURE 2:**
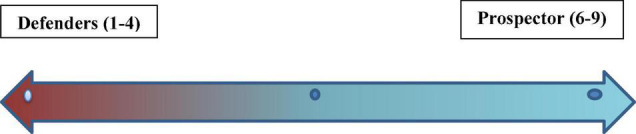
The continuum of the business strategy.

Three ratios, MESR, COGSR, and CASGR, are used for scoring the strategic orientation. Thus, to calculate the composite score, the whole data has been divided among three major groups that are underscored with different values as firms with the most significant points of ratios given a score of 3. Firms with moderating points were given a score of 2, and firms with the lowest points of proportions were given a score of 1. The summary scores are 9 representing the maximum score, and 3 showing the minimum score a company could receive. The measure of business strategy is used in this study as business strategy (BSSTRA) used as a dummy variable where 1 shows the prospector nature of the firm and 0 denotes the defender strategy of companies. To arrange the discrete scores along a continuum strategy type has a category of prospector. Figure 2 depicts a strategy continuum representing discrete scores ranging from 1 to 9. The continuum has two ends, one representing a score from 1 to 4 and the other representing a score from 5 to 9. Furthermore, companies with a value between 6 and 9 are considered prospectors, whereas others are not.

#### Number of Female Director in Board

Prior researches use numerous measures for measuring board diversity. Beji et al. (2021) have used female directors on the board as a dummy variable, and its value is equal to 1. Some studies also used the Blau-index, which measures evenness in the distribution of the men and women on the board of directors (Amorelli and García-Sánchez, 2021). Following (Khaoula and Moez, 2019), this study measures gender diversity as a percentage of the board’s female directors (members) (Figure 1). The formula for measurement IS:


$$\text{FD}=\frac{\mathrm{Number}\,\mathrm{of}\,\mathrm{Women}\,\mathrm{in}\,\mathrm{Board}}{\text{To}\,\mathrm{tal}\,\mathrm{Number}\,\mathrm{of}\,\mathrm{Direc}\,\text{to}\,\mathrm{rs}\,\mathrm{on}\,\mathrm{Board}}$$

#### Profitability

The ability of a corporation to make profits large enough to generate revenue from year to year is the measure of its profitability. Companies with a greater return on assets (profitability) have a higher ETR than those with lower assets (Jaffar et al., 2021). The return on assets (ROA) can be calculated with the following ratio:


$$\text{ROA}=\frac{\mathrm{Net}\,\mathrm{Income}}{\text{To}\,\mathrm{tal}\,\mathrm{assets}}$$

#### Firm Size

The total amount of assets possessed by a corporation can be used to determine the size of a company. Other researchers have utilized the natural logarithm of a corporation’s total assets to determine the company’s size. This study also uses this formula to measure the firm size (Ullah, 2012; Aslam and Ullah, 2017; Ali et al., 2021).


$$\mathrm{Firm}\,\mathrm{Size}={\text{LTA}}_{\text{t}}=\ln\left(\text{To}\,\mathrm{tal}\,\mathrm{Assets}\right)$$

#### Leverage

DER is a proxy used for denoting leverage, and it can be measured as long-term debt scaled by the total assets. The cost of interest that arises from long-term debt is a decrease in a company’s gross income. When the interests costs are higher, it will reduce firms’ tax burdens (Wang et al., 2020). This thing positively affects a firm and then acts as a motivational tool and incentive for a firm to carry out its tax planning (Chen et al., 2010). This research uses the measurement that the previous studies have used to measure leverage (Sarwar et al., 2018).


$$\text{DER}=\frac{\mathrm{Debt}}{\text{To}\,\mathrm{tal}\,\mathrm{Equity}}$$

### Generalized Method of Moment

To investigate whether Business Strategy and corporate governance moderation effect influence the company’s decisions related to firms behavior regarding tax management. We used GMM for measuring these relationships among variables. According to the literature, indigeneity bias and the issue of the omitted variable are two types of inconsistencies that are likely to be present in the majority of empirical work on tax policy that is now available. Because of the nature of OLS, the lag in tax avoidance is likely to be connected with firm-specific factors, resulting in inconsistent and biased findings when the variable is utilized. First and foremost, this work shows how these two biases affect panel data estimators when both tendencies are considered. As a result of the biases we discovered, we used GMM estimators to get our forecasts. In order to correctly analyze the dividend policy, it is required to first investigate the dynamics of the dividend policy in order to be able to analyze it and overcome any potential issues that may result from the use of panel data estimator methodologies. Because a firm’s tax policy changes over time, non-financial firms benefit more from integrating cross-sectional and time-series data. Furthermore, time-series data can disclose additional potential information that cross-sectional data may ignore. It also considers firm-specific effects and regression indigeneity, among other factors.

To examine the effect of female directors on a board (FD) as characteristics of corporate governance and for testing the impact of business strategy (BSTRAT) in the presence of female directors on tax avoidance, the following regression model has been developed:


(1) $$G\,A\,A\,P\,\_\,E\,T\,R_{t}=\beta_{0}+\beta_{1}\,B\,S\,T\,R\,A\,T_{t}+\beta_{2}\,F\,D_{t}+\beta_{3}\,B\,S\,T\,R\,A\,T_{t}*F\,D_{t}+\beta_{4}\,L\,T\,A_{t}+\beta_{5}\,R\,O\,A_{t}+\beta_{6}\,D\,E\,R_{t}+\varepsilon_{t}$$

Here, GAAP-ETR = measure of tax avoidance, BSTRATT = business strategy, BS = Board Size.

FD = (# of female directors in a board), LTN = log of total assets a proxy for firm size measurement, DER = Debt to equity ratio a proxy for leverage measurement, ROA = proxy for profitability.

The method of moments is used to estimate population parameters in statistics. It is a generic strategy used in semiparametric models when the parameters of interest are in a finite dimension, and the shape of the data distribution is unknown. A model requires a set of moment conditions that are functions of inputs and model parameters. The GMM then minimizes the samples averages’ particular norms, making it a special case of minimal distance estimation. For identified models, the number of moment conditions exceeds the parameter dimension vector. Sargan test was proposed by Sargan (1958).

## Empirical Results

This segment shows the results of descriptive analysis of dependent variable (ETR) moderators (FD) and control variables (LTA, ROA, and DER). The study applied descriptive statistics to examine the central distribution of tax avoidance. Table 1 summarizes these statistics that comprise of the mean, standard deviation of variables, and minimum and maximum values. According to these statistics, the tax rate’s mean value and standard deviation are 0.1800 and 0.1772. Respectively, these results are consistent with previous research on developing economies (Aburajab et al., 2019). The variable for the existence of female directors onboard has an average of 1.0688 and a standard deviation of 1.1860. The average value and standard deviation of firm size are 15.4331 and 1.0145, respectively, consistent with various researchers (Martinez and Ferreira, 2019). The mean value and standard deviation of profitability are 0.1398 and 0.1255, respectively, according to the existing literature (Abbas et al., 2019d; Aqeel et al., 2021; Farzadfar et al., 2022; Ge et al., 2022; Rahmat et al., 2022). Furthermore, the mean and standard deviation value of leverage is 0.6242 and standard deviation is 0.4565, also the maximum level of this ratio is 1.0046 and the minimum level is 0.0914.

**TABLE 1:** Descriptive feature.

Variables	Average	*SD*	Min	Max
ETR	0.1800	0.1772	–0.2520	0.8006
FD	1.0688	1.1860	0	4
LTA	3.6583	0.6044	13.3551	17.2203
ROA	0.1398	0.1255	–0.6417	0.7882
DER	0.6242	0.4565	0.0914	1.0046

*ETR; GAAP_ETR (a proxy for measuring tax avoidance), FD; Female Director in Board, LTA; Firm Size, ROA; profitability, DER; Leverage. SD shows the standard deviation, Min and Min offers the minimum and maximum value of the variables.*

The association between a dependent variable, an independent variable, and a control variable is represented by a correlation matrix. This matrix denotes how much change in one variable can change other relative variables. Table 2 describes the correlation matrix of this study. Firm ROA and DER are substantially indirectly associated, which indicates that their relative movements are opposed to one another. GAAP_ETR are highly negatively correlated and female directors on board.

**TABLE 2:** Correlation table.

	ETR	FD	LTA	ROA	DER
ETR	1				
FD	0.0761	1			
LTA	0.1363	0.2129	1		
ROA	−0.0555	−0.1086	0.0846	1	
DER	−0.0305	−0.0195	−0.1044	−0.3305	1

### Generalized Moment Method Estimation

We have used the GMM to examine our hypothesis. Equation no. 1 is estimated by following the GMM technique by following the literature (Minnick and Noga, 2010). In the class of all the estimators that do not use any type of extra information aside from that contained in the moment conditions, the estimators of GMM are known to be asymptotically regular, efficient, and consistent.

Table 3 shows the results of the estimated models. The value of L1 shows no effect on tax payments. Business strategy as an independent variable has a significant negative relation (*p* = 0.04). Female directors’ value offers a positive and insignificant connection with tax rates. Moderate factor (FDBSTRATT) is significantly and positively associated. The size of the board is highly similar to tax payments, and this relationship has a negative value. Company ROA and DER are found to be inversely associated with tax payments, whereas the firm size is found to be directly associated with tax payments.

**TABLE 3:** The moderating role of female directors on board.

	GAAP_ETR		
	Coefficient	SE	*p*-value
L1	–0.2408	0.0318	0.125
BSTRATT	–0.0696	0.0190	0.04
FD	0.0567	0.0112	0.324
FD * BSTRATT	0.0128	0.0126	0.045
LTA	0.1124	0.0226	0.08
ROA	–0.0065	0.0014	0.05
DER	–0.0477	0.0193	0.009
Constant	2.0720	0.3510	0
Sargan test (*p*-value)	0.15	0.23	0.13

## Discussion

The findings indicate a positive relationship between corporate strategy and tax avoidance. This study suggests that the BSTRAT (prospector strategy trend in businesses) is more robust, the lower the tax payments will be in the future. This expression implies that organizations that employ a prospector strategy are inclined to save supplementary money on taxes than their counterparts. The findings of this study are congruent with those of prior investigations (Higgins et al., 2015; Hsu et al., 2018; Martinez and Ferreira, 2019). It is because the prospector generates a high level of income from a considerable share of the total market as well as from the sale of their creative items, which makes the cost of tax avoidance for them insignificant. Due to these strengths of prospectors’ tax avoidance, costs have no significant effect on their survival (Higgins et al., 2015; Sadjiarto et al., 2020). Findings corroborate hypothesis H1, which states that “company strategy has a major influence on tax avoidance.”

Concerning the results of corporate governance characteristics, the presence of female directors onboard has no significant impact on tax avoidance. This effect can be two reasons: a negligible proportion of female directors on board and the male dominance in decision-making regarding tax planning. Results are consistent with the previous study (Khaoula and Ali, 2012). Results of female directors as moderators with business strategy FDBSTRAT show significant and positive relation with tax payment and explain how the inclusion of female directors in prospector enterprises will reduce the use of tax avoidance techniques. These findings support hypothesis 3 of the study, which states that “the existence of female directors considerably moderates the association among corporate strategy and tax.”

The findings of the company size test show a positive sign, indicating that the greater the size of a firm, the higher the amount of tax paid and the lower the amount of tax avoided. It aligns with political cost theory, which holds that giant corporations seek to avoid a poor reputation while maximizing financial success. Results align with (Minnick and Noga, 2010; Boussaidi and Hamed, 2015; Martinez and Ferreira, 2019). The profitability results are indirect and imply as ROA improves, tax payments would drop (Pratama, 2017). This study’s findings show a link between leverage and tax evasion. These findings support earlier studies showing greater DER businesses had lower ETR (Higgins et al., 2015; Sadjiarto et al., 2020). Above findings of control variables and their discussion support H5, H6, and H7 of this study.

## Conclusion of the Study

This research aimed to link corporate governance, commercial strategy, and tax evasion. Furthermore, this study examines how business strategy influences corporate tax evasion actions when female directors are present. Based on a sample of 115 companies listed on the Pakistan stock exchange, this study employed the GMM (generalized method of moments) technique for testing the hypothesis. The data was selected from different non-financial sectors based on the availability of enough data. The period of data is 5 years—2013–2017. We use GAAP_ETR as a proxy of tax avoidance acting as the dependent variable and develop a dummy variable to measure the business strategy. In contrast, corporate governance characteristics act as moderators between the dependent and independent variables.

According to the findings of this study, company strategy has a statistically significant and negative relationship with tax evasion, which suggests that enterprises with prospector-type strategies are further probable to engage in tax management. Because of the moderating influence of corporate governance, tax avoidance actions of prospector-type enterprises produce diverse outcomes conditional on the characteristics of the moderating variable being used, as shown in Table 4. For example, the inclusion of women on the board will reduce the tax avoidance activities of prospector enterprises. On the contrary, bigger board size is associated with decreased tax payments. Also, the prospector firms’ tax avoidance actions will be curtailed due to board independence.

**TABLE 4:** Sample reconciliation.

Details	Sample	Observations
Total sample from non-financial firms listed on Pakistan Stock Exchange (2013–2017)	138	690
Delisted firms from the sample which not have data of at least two measures being used	(23)	(115)
The total sample used for analysis	115	575

This study extends the existing literature on various dimensions (Minnick and Noga, 2010; Richardson et al., 2013; Armstrong et al., 2015; Higgins et al., 2015). Second, this study also contributes to the literature. Most previous studies determine the relation between tax avoidance activities and corporate governance determinants and features, and others find the connection between tax avoidance and business strategies. To our knowledge, this study is the first to examine the role of corporate governance traits as a moderator in tax management and company strategy in Pakistan.

According to the conclusions of this study, financial regulators must encourage prospector enterprises to boost the number of female directors on their boards of directors. According to the findings of this study, the inclusion of female directors on boards of directors has the potential to minimize tax avoidance operations in corporations. This study uses time-series data analysis, and the sample is taken from a small pool of data. The workings and findings of this study suggest that depending on the context of other types of corporate governance, such as board independence, board structure, audit committee, remunerations, and so on, should be used as moderators with different realms and association with tax avoidance and business plans in both advanced and emerging economies. In future investigations, panel data can be employed as a sampling method. In future studies, it may be required to apply more than three corporate strategy ratios in order to determine the influence of tax evasion on various dimensions in both emerging and developed countries.

Before generalizing such forecasts, it is crucial to consider the particular characteristics of each region, such as the ease with which a country can access the capital market, the tax-deductibility of R&D investments, and legal issues. This study proposes that we investigate the strategic types of organizations to establish the impact of strategy on aggressive tax planning in specific industries and the deployment of aggressive strategies by defense enterprises in other areas of management.

## Data Availability Statement

The original contributions presented in the study are included in the article/supplementary material, further inquiries can be directed to the corresponding author/s.

## Author Contributions

XZ and RZ significantly provided major contributions to revising this manuscript and provided contribution to resources to make possible this manuscript. JA conceptualized the idea, contributed to the study design, completed the entire article, including introduction, literature, discussion, and conclusion, and edited the original manuscript before submission. All authors contributed to the literature, discussion, and conclusion and reviewed and approved the final edited version for publication.

## Conflict of Interest

The authors declare that the research was conducted in the absence of any commercial or financial relationships that could be construed as a potential conflict of interest.

## Publisher’s Note

All claims expressed in this article are solely those of the authors and do not necessarily represent those of their affiliated organizations, or those of the publisher, the editors and the reviewers. Any product that may be evaluated in this article, or claim that may be made by its manufacturer, is not guaranteed or endorsed by the publisher.
